# Editorial: Immune response in EBV infection: from persistence to viral-associated tumours

**DOI:** 10.3389/fimmu.2026.1816022

**Published:** 2026-03-06

**Authors:** Paola Chabay, Jianmin Zuo

**Affiliations:** 1 Laboratory of Molecular Biology, Pathology Division, Multidisciplinary Institute for Investigation in Pediatric Pathologies (IMIPP-CONICET-GCBA), Ricardo Gutiérrez Children’s Hospital, Buenos Aires, Argentina; 2 School of Infection, Inflammation and Immunology, College of Medicine and Health, University of Birmingham, Birmingham, United Kingdom

**Keywords:** children, EBV, immune response, lymphoma, multiple sclerosis

Since its discovery in 1964 by Anthony Epstein and his co-workers Yvonne Barr and Bert Achong (1), the Epstein Barr virus (EBV) has drawn the attention of tumor virologists, due to its unique biological behavior. In most individuals, the virus infects the host and establishes a latent infection for life as a harmless passenger, maintaining a delicate and dynamic balance with the immune system of healthy carriers (2). While primary infection, particularly in children is often clinically silent (3), viral persistence depends on finely tuned immune surveillance, and its dysregulation may culminate in chronic inflammation, autoimmunity, or malignancy (4). There is a complex interplay between EBV-related pathologies and immune response, often reflecting an imbalance in this equilibrium (5–9).

The contributions gathered in this Research Topic illustrate the long way from the first stage of EBV infection through the development of EBV-associated diseases that disrupts the balance between the virus, ultimately advancing our understanding of these conditions. Together, these five studies expand our understanding of immune heterogeneity across the life course, antiviral antibody landscapes, micronutrient-immune-virus interactions, inborn errors of immunity predisposing to EBV pathology, and the spatial organization of the tumor microenvironment in EBV-driven lymphomas.

In this Research Topic, Nalwoga et al. provide a population-level immunological framework for interpreting EBV persistence in endemic settings. By characterizing age-associated changes in innate and adaptive immune compartments, the authors reveal that immune architecture is dynamically remodeled across the lifespan under the influence of chronic pathogen exposure. In a region where EBV infection occurs early in life and coexists with multiple environmental challenges, shifts in T cell differentiation states, activation markers, and functional capacity may influence antiviral control. Age-related immune remodeling may therefore determine the balance between latent infection and pathological reactivation.

Vasilenko et al. analyzed the qualitative features of humoral immunity in the context of EBV-associated autoimmunity. Serological mapping demonstrated an altered immunological response to EBV in multiple sclerosis (MS) patients, suggesting a potential role for EBNA1 as an antigenic driver. These findings illustrate a critical intermediary stage between benign persistence and overt malignancy: dysregulated immune responses to EBV, potentially creating a pro-inflammatory environment that predisposes to EBV-related disease.

Expanding on host-environment interactions, Rasheed et al. discussed how three well-recognized risk factors, EBV infection, hypovitaminosis D, and dysregulated immune system, interact in the pathogenesis of MS. The authors synthesize evidence that vitamin D deficiency may weaken cytotoxic T cell control of EBV-infected B cells while simultaneously promoting pro-inflammatory responses.

The genetic aspect of EBV susceptibility is directly addressed by del Pino Molina et al., who performed a functional validation of two variants in the MAGT1 gene, associated with XMEN disease, and demonstrated how perturbations in magnesium transport and glycosylation pathways reshape B cell subsets and cellular phenotypes, thereby creating a permissive niche for uncontrolled EBV-driven B cell proliferation.

Finally, Qian et al., using spatial transcriptomics and multiplex immunofluorescence, delineated striking differences between EBV-positive nodal T/NK-cell lymphoma (EBV+ nTNKL) and extranodal NK/T-cell lymphoma (ENKTL). ENKTL exhibits features of an immune-desert phenotype with high malignant cell density and neutrophil-associated signaling, whereas EBV+ nTNKL demonstrates a more immune-active microenvironment enriched in cytotoxic T cells, B cells, and PD-1/PD-L1 expression. This work illustrates how advanced spatial technologies can decode the complexity of the tumor microenvironment of EBV-driven cancers and guide subtype-specific immunotherapeutic strategies.

Taken together, the contributions to this Research Topic describe diverse dimensions of EBV–host interactions. EBV-associated diseases cannot be understood solely through viral genetics or host immunity in isolation. Rather, it is the dynamic interplay between persistent infection, immune regulation, genetic background, and tissue context that determines clinical outcomes.

## References

[1] Epstein MA Achong BG Barr YM . Virus particles in cultured lymphoblasts from burkitt’s lymphoma. Lancet. (1964) 283:702–3. doi: 10.1016/S0140-6736(64)91524-7, PMID: 14107961

[2] Rostgaard K Balfour HH Jr Jarrett R Erikstrup C Pedersen O Ullum H . Primary Epstein-Barr virus infection with and without infectious mononucleosis. PLoS One. (2019) 14:e0226436. doi: 10.1371/journal.pone.0226436, PMID: 31846480 PMC6917282

[3] Chabay PA Preciado MV . EBV primary infection in childhood and its relation to B-cell lymphoma development: a mini-review from a developing region. Int J Cancer. (2013) 133:1286–92. doi: 10.1002/ijc.27858, PMID: 23001576

[4] Zhong LY Xie C Zhang LL Yang YL Liu YT Zhao GX . Research landmarks on the 60th anniversary of Epstein-Barr virus. Sci China Life Sci. (2025) 68:354–80. doi: 10.1007/s11427-024-2766-0, PMID: 39505801

[5] Long HM Taylor GS . The T cell response to epstein-barr virus. In Curr Top Microbiol Immunol. Springer, Berlin, Heidelberg. (2026). doi: 10.1007/82_2025_339, PMID: 41649523

[6] Läderach F Bremer E Münz C . Latent, lytic, and linked to multiple sclerosis-how EBV drives autoimmunity. Eur J Immunol. (2026) 56:e70153. doi: 10.1002/eji.70153, PMID: 41723724 PMC12925384

[7] Bednarska K Chowdhury R Tobin JWD Swain F Keane C Boyle S . Epstein-Barr virus-associated lymphomas decoded. Br J Haematol. (2024) 204:415–33. doi: 10.1111/bjh.19255, PMID: 38155519

[8] Mangiaterra T Alonso-Alonso R Rabinovich A De Dios Soler M Galluzzo L Soria M . Presence of Epstein-Barr virus (EBV) antigens detected by sensitive methods has no influence on local immune environment in diffuse large B cell lymphoma. Cancer Immunol Immunother. (2024) 73:29. doi: 10.1007/s00262-023-03617-x, PMID: 38280007 PMC10821829

[9] Zuo J Jin DY . Immune evasion by epstein-barr virus. Curr Top Microbiol Immunol. (2025). doi: 10.1007/82_2025_311, PMID: 40705087

